# Race, pigmentation, and the human skin barrier—considerations for dermal absorption studies

**DOI:** 10.3389/ftox.2023.1271833

**Published:** 2023-10-11

**Authors:** Alec T. Salminen, Prashiela Manga, Luísa Camacho

**Affiliations:** ^1^ U.S. Food and Drug Administration, National Center for Toxicological Research, Jefferson, AR, United States; ^2^ U.S. Food and Drug Administration, Office of Cosmetics and Colors, Center for Food Safety and Applied Nutrition, College Park, MD, United States

**Keywords:** race, pigmentation, skin, dermal absorption, skin barrier models

## Abstract

A functional human skin barrier is critical in limiting harmful exposure to environmental agents and regulating the absorption of intentionally applied topical drug and cosmetic products. Inherent differences in the skin barrier between consumers due to extrinsic and intrinsic factors are an important consideration in the safety assessment of dermatological products. Race is a concept often used to describe a group of people who share distinct physical characteristics. The observed predisposition of specific racial groups to certain skin pathologies highlights the potential differences in skin physiology between these groups. In the context of the human skin barrier, however, the current data correlating function to race often conflict, likely as a consequence of the range of experimental approaches and controls used in the existing works. To date, a variety of methods have been developed for evaluating compound permeation through the human skin, both *in vivo* and *in vitro*. Additionally, great strides have been made in the development of reconstructed human pigmented skin models, with the flexibility to incorporate melanocytes from donors of different race and pigmentation levels. Together, the advances in the production of reconstructed human skin models and the increased adoption of *in vitro* methodologies show potential to aid in the standardization of dermal absorption studies for discerning racial- and skin pigmentation-dependent differences in the human skin barrier. This review analyzes the existing data on skin permeation, focusing on its interaction with race and skin pigmentation, and highlights the tools and research opportunities to better represent the diversity of the human populations in dermal absorption assessments.

## 1 Introduction

### 1.1 Race and skin type

Race is a concept often used to describe a group of people who share distinct physical characteristics, such as the color of their skin. Ethnicity, alternatively, differentiates among groups of people according to their cultural expression and identification. In practice, race and ethnicity are generally associated with a myriad of intrinsic (e.g., genetic) and extrinsic (e.g., culture and socioeconomics) factors (Lu et al., 2022). In the context of pharmacology/toxicology, one’s race/ethnicity can be considered a determinant in response to medical treatment. A 2015 review conducted by the United States Food and Drug Administration (US FDA) found that approximately one-fifth of new drugs approved between 2008 and 2013 showed differences in exposure and/or response across racial/ethnic groups (Ramamoorthy et al., 2015). Furthermore, minorities have been historically under-represented in clinical trials and basic science research in the United States (Oh et al., 2015). Together, these realities highlight the importance in considering race and ethnicity as variables in regulatory and basic science research, and the need for studies to represent adequately the population diversity.

Given the complexity surrounding the biological definition of one’s race/ethnicity, the US FDA has provided guidance to help standardize the collection of race and ethnicity data across clinical trials (U.S. Food and Drug Administration, 2016). The recommended standardized approach is based on the Office of Management and Budget’s (OMB) Policy Directive No. 15 (Office of Management and Budget, 1997), which provides five minimum categories for data on race, namely, 1) American Indian or Alaska Native, 2) Asian, 3) Black people or African American, 4) Native Hawaiian or other Pacific Islander, and 5) White people, along with two categories for data on ethnicity, namely, 1) Hispanic or Latino and 2) Not Hispanic or Latino. Of note, the Chief Statistician of the U.S. announced recently a formal review to revise the OMB Policy Directive No. 15 (Orvis, 2022). With continued improvement, guidelines can help harmonize racial/ethnic data collection in research and clinical trials. Although the use of race/ethnicity is useful, it is important to bear in mind the confounding factors associated with their use. For example, self-reported race/ethnicity is prone to bias, and population stratification in ethnically admixed populations is well-documented (Mersha and Abebe, 2015).

With skin color dominating the current societal designations of race, skin pigmentation is often the key focus of studies on race and human physiology (Jablonski, 2021b). Pigmentation of human skin is directly regulated by the function of specialized dendritic cells termed melanocytes (Cichorek et al., 2013). Skin melanocytes primarily reside in the epidermis at the epidermal–dermal junction flanking keratinocytes (Kolarsick et al., 2011). By the process of melanogenesis, melanocytes produce melanin, the chemical responsible for skin pigmentation, in lysosome-related organelles called melanosomes (Orlow, 1995). Genetics (Jablonski, 2021a; Feng et al., 2021); melanin quantity and type (Alaluf et al., 2002); melanosome quantity, size, and distribution (Thong et al., 2003); and the ability to respond to UV light exposure (Maddodi et al., 2012) all drive observed differences in skin pigmentation and, thus, skin color across race.

To facilitate the execution of studies concerning skin pigmentation, criteria for the classification of skin pigmentation levels have been established. In 1975, the Fitzpatrick skin phototype scale was created to provide a basis on which appropriate dosing of oral methoxsalen could be determined (Gupta and Sharma, 2019). Although the original skin classifications, ranging from Fitzpatrick type I to IV, were based on Caucasian skin and its native reaction to sun exposure, the scale has since been updated to include two additional skin types, V and VI, to better represent all individuals (Fitzpatrick, 1988). One shortcoming of the Fitzpatrick scale, however, is that the classifications are based on self-reporting and thus can be subjected to bias (Okoji et al., 2021). A more objective method to determine skin pigmentation is the individual typology angle (ITA) measurement of constitutive pigmentation, which ranges from 55° for very light skin to −30° for dark skin. The ITA is calculated based on two variables, luminance and the yellow to blue color of the skin, both of which can be determined by non-invasive, commercially available instrumentation (Osto et al., 2022). Importantly, race and skin pigmentation do not follow a strict correlation. Thus, the Fitzpatrick scale and ITA may be used in conjunction with OMB’s racial/ethnic classifications to categorize better patients and patient samples (Stamatas et al., 2004).

### 1.2 Overview of skin permeation studies

Understanding how the human skin barrier functions to limit or permit the passage of specific compounds is of interest to consumers and regulators alike. Dermal absorption of hazardous substances is considered one of the key routes of toxicity during incidental occupational and environmental exposures (Schneider et al., 1999; Franken et al., 2015b). Furthermore, assessing the rate and amount of skin permeation of topical drugs and cosmetics is critical in evaluating their safety and, in the case of topical drugs, efficacy (Nohynek et al., 2010; Chevillotte et al., 2014; Raney et al., 2015). In clinical settings, human skin permeation kinetics can be determined through a set of established methods. These can include tape stripping, in which layers of the topically exposed *stratum corneum* are sequentially removed by using a tape and analyzed for compound content; dermal microdialysis, where a probe is inserted near the dermal capillary bed to measure compound levels and estimate permeation; or systemic analyses, where urine and/or blood are periodically sampled, following dermal exposure (Polak et al., 2012; Raney et al., 2015). Pre-clinical studies of dermal absorption may also be performed, both *in vivo* with animal models and *in vitro* (Jung and Maibach, 2015). *In vitro* permeation testing (IVPT), a skin barrier, is mounted on either a static Franz diffusion cell (Franz, 1975) or a flow-through Bronaugh-type diffusion cell (Bronaugh and Stewart, 1985), and the apical surface of the model is exposed to the compound of interest (Santos et al., 2020).

## 2 Current perspectives on the correlation of race and the human skin barrier

**FIGURE 1:**
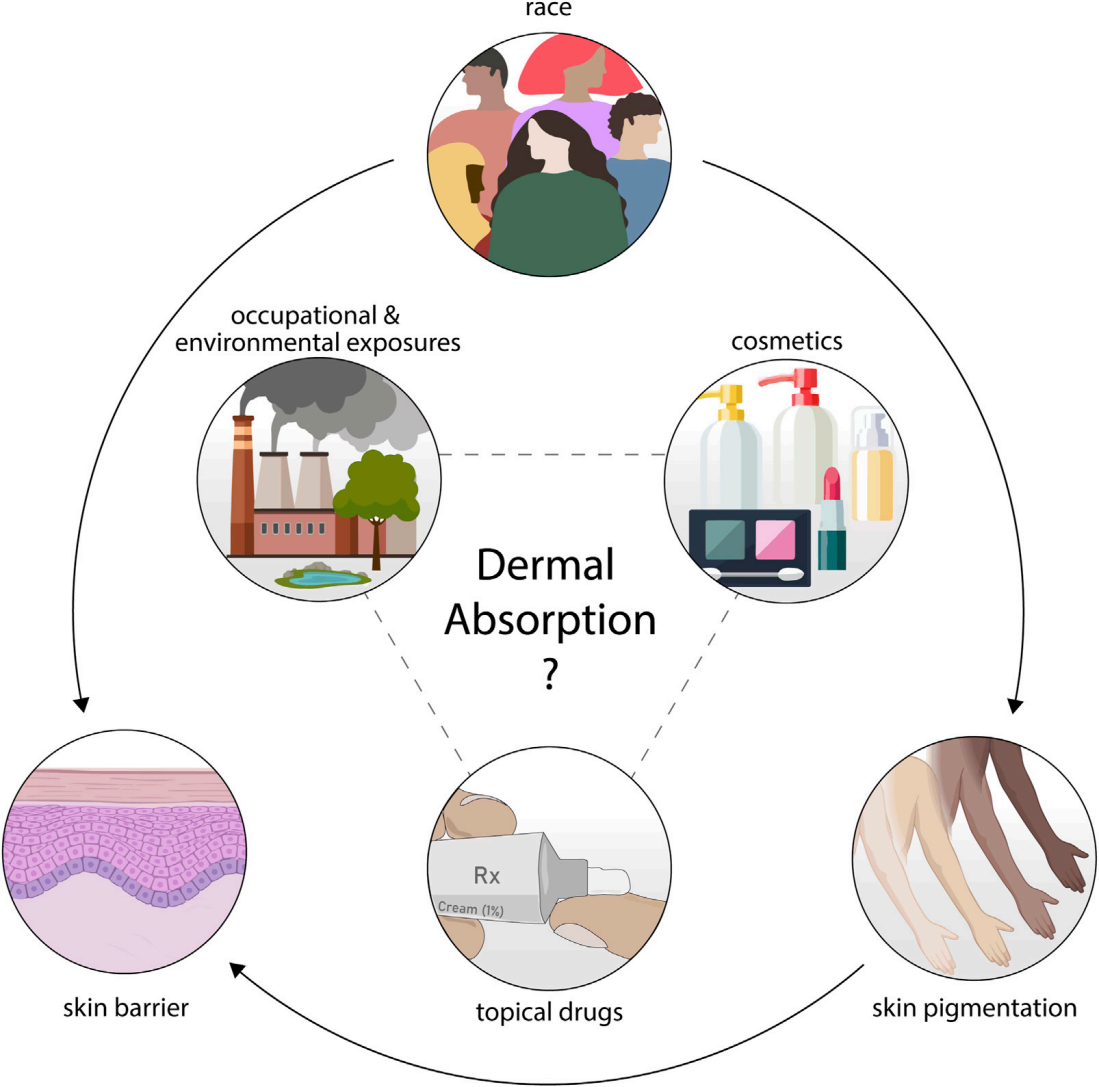
Interest in the interplay between race, skin pigmentation, and the human skin barrier has driven research in this context. A consensus understanding of how these variables relate and modulate the dermal absorption of topical drugs, cosmetics, or occupational and environmental exposures remains to be elucidated.

Although studies aimed at relating race and the human skin barrier exist, they often lack consensus in their findings (Alexis et al., 2021). In addition, mechanistic studies on the biological factors regulating skin permeation across skin types are limited. The consequential gap in knowledge is to the detriment of safety assessors as it is still unclear whether one’s race may predispose an individual to a higher level of toxicity via dermal absorption (Figure 1).

### 2.1 The *stratum corneum*

Several studies have investigated the differences in the *stratum corneum*, the apical layer of the skin primarily responsible for the dermal barrier, across race (Table 1). In a study by Weigand and others, a cohort of Black and White people's skin sections was collected and processed with methylene blue staining for the visualization of the *stratum corneum* (Weigand et al., 1974). The authors reported consistent *stratum corneum* thickness across the racial groups investigated but observed that the Black cohort had more corneocyte layers. These results were in line with their additional findings that the skin from the Black cohort required more tape strips to completely remove the *stratum corneum* when compared to the skin from the White cohort. Together, these data suggest the *stratum corneum* of the Black cohort was denser when compared to that of the White cohort. In a more recent study, racial differences in the *stratum corneum* were again probed, this time focusing mainly on the cellular constituent itself, the corneocytes (Corcuff et al., 1991). Corneocytes were collected from the Black, White, and Asian subjects via standardized methods and processed for visualization and size quantification. The authors found that there were no significant differences in the corneocyte surface area across the racial groups but that the skin of the Black subject group had a significantly higher rate of spontaneous desquamation (i.e., shedding) when compared to the skin of the Asian and White subject groups. Of note, corneocyte collection was performed on a partially sun-protected portion of the upper arm to limit the influence of UV light exposure on desquamation results. Work by Warrier and others found opposite results when desquamation was quantified on the cheeks and foreheads of Black and White individuals (Warrier et al., 1996), highlighting the influence of skin location on the phenotype.

**TABLE 1:** Summary of highlighted studies on race and the human skin barrier.

Comparison	Reference	Subject/donor race (n)	Relevant findings
*Stratum corneum* physiology	Weigand et al. (1974)	Black (10–17)	No difference in the mean *stratum corneum* thickness across Black (6.5 µm, n = 17) and White (7.2 µm, n = 18) subjects (mean); Significantly higher (*p* < 0.01; Mann–Whitney U test) mean number of cell layers in Black (21.8, n = 10) *vs*. White (16.7, n = 10) subjects *stratum corneum* (mean)
		White (10–18)	
	Corcuff et al. (1991)	Black (18–25)	No difference in the mean corneocyte surface area across Black (911 ± 20 μm^2^), White (899 ± 22 μm^2^), and Asian (909 ± 24 μm^2^) subjects (mean ± standard error); 2.5 times higher (*p* < 0.001) mean spontaneous desquamation observed in Black (26,500 ± 4,900 per cm^2^) *vs.* White (11,800 ± 1,700 per cm^2^) and Asian (10,400 ± 2,100 per cm^2^) subjects (mean ± standard error)
		White (18–25)	
		Asian (18–25)	
	Warrier et al. (1996)	Black (30)	Significantly higher (*p* < 0.05; two-tailed Student’s t-test) mean desquamation index on White subjects’ cheeks and foreheads compared to Black subjects’ cheeks and foreheads, but no differences across races when measured on subjects’ legs (comparisons within anatomical groups)
		White (30)	
	Muizzuddin et al. (2010)	Black (73)	Significantly fewer (*p* < 0.001; ANOVA with *post hoc* Tukey test) mean C18 phytosphingosine-based ceramides (normalized to total ceramides) in Black (0.74 ± 0.25 µg/mg) *vs.* White (1.18 ± 0.46 µg/mg) and Asian (1.14 ± 0.51 µg/mg) donor *stratum corneum* (mean ± standard deviation)
		White (119)	
		Asian (149)	
	Jungersted et al. (2010)	Black (18)	Significantly lower (*p* < 0.001; two-tailed Mann–Whitney test with Bonferroni correction) mean ceramide/:cholesterol ratio in Black (0.8) *vs.* White (1.7) and Asian (2.1) donor *stratum corneum* (mean)
		White (28)	
		Asian (25)	
	Berardesca et al. (1998)	Black (8)	No difference in the mean baseline *stratum corneum* pH, but significantly higher (*p* < 0.05; Student’s t-test) mean *stratum corneum* pH after three tape strips (TSs) in White (5.0 ± 0.7 (baseline) and 4.8 ± 0.3 (three TSs) *vs.* Black (4.8 ± 0.3 (baseline) and 4.3 ± 0.3 (three TSs) subject skin (mean ± standard deviation)
		White (10)	
	Young et al. (2019)	Black (17)	Significantly higher (*p* < 0.0001, r_s_ = 0.71; Spearman rank correlation) mean skin surface pH in Black *vs.* White donors
		White (27)	
Skin transepidermal water loss (TEWL)	Berardesca et al. (1988)	Black (10)	No difference in the mean basal TEWL between Black (3.3 ± 0.9 g/h) and White (2.7 ± 0.7 g/h) subjects (mean ± standard error)
		White (9)	
	Berardesca et al. (1998)	Black (8)	No difference in the mean basal TEWL between Black and White subjects but significantly higher (*p* < 0.05; Student’s *t*-test) mean TEWL in Black *vs.* White subjects after three and six tape strips (TSs; mean ± standard deviation; g/m^2^ h): Black subjects = 7.7 ± 0.9 (basal), 10.5 ± 1.7 (three TSs), and 12.3 ± 3.2 (six TSs); and White subjects = 7.3 ± 0.9 (basal), 8.8 ± 1.7 (three TSs), and 9.7 ± 1.4 (six TSs)
		White (10)	
	Reed et al. (1995)	Black (4)	No difference in the mean basal TEWL between Asian (5.1 ± 0.6 g/m^2^ h) and White (4.8 ± 0.4 g/m^2^ h) individuals nor individuals divided based on Fitzpatrick skin type (II/III *vs.* V/VI)
		White (8)	
		Asian (6)	
	Voegeli et al. (2015)	Black (not specified, 60 total subjects)	No difference in the mean basal TEWL across Black and White individuals but significantly higher (*p* < 0.01; ANOVA with *post hoc* Tukey tests) mean basal TEWL observed in African subjects with albinism *vs.* pigmented African subjects
		White (not specified, 60 total subjects)	
	Wilson et al. (1988)	Black (10)	Significantly higher (*p* < 0.01) mean TEWL for Black (2.79 µg/cm^2^/h) *vs.* White (2.61 µg/cm^2^/h) donor skin, as averaged across five skin temperatures (20, 25, 30, 35, and 40°C; mean)
		White (12)	
	Warrier et al. (1996)	Black (30)	Significantly higher (*p* < 0.05; two-tailed Student’s t-test) mean TEWL on the cheeks and legs of White *vs.* Black subjects (no difference on forearms)
		White (30)	
Skin permeation	Williams et al. (1991)	Black (4)	Significantly higher (*p* < 0.05; ANOVA) mean maximum level of nitroglycerin metabolites 1,2-glyceryl dinitrate and 1,3-glyceryl dinitrate in White/Asian subjects (2.72 ± 0.74 ng/mL and 0.78 ± 0.30 ng/mL, respectively) compared to that of Black subjects (1.69 ± 0.96 ng/mL and 0.0.41 ± 0.13 ng/mL, respectively), following topical exposure (mean ± standard deviation)
		White/Asian (12)	
	Lotte et al. (1993)	Black (6–9)	No difference in the mean/predicted urine concentration nor *stratum corneum* content of benzoic acid, caffeine, or acetylsalicylic acid between all racial groups tested, following topical exposure
		White (6–9)	
		Asian (6–9)	
	Franken et al. (2015a)	Black (3)	Significantly higher (*p* ≤ 0.05; ANCOVA with *post hoc* Bonferroni test) mean 24-h cumulative permeation of platinum (delivered as potassium tetrachloroplatinate) through Black (37.52 ± 10.61 ng/cm^2^) *vs.* White (5.05 ± 1.54 ng/cm^2^) donor skin, as measured in a Franz diffusion cell (mean ± standard error)
		White (3)	

Studies on lipid content (Jungersted et al., 2010; Muizzuddin et al., 2010) and pH (Berardesca et al., 1998; Young et al., 2019) further suggest potential differences in the *stratum corneum* across racial groups. Works by Muizzuddin et al. and Jungersted et al. reported reduced ceramide or ceramide:cholesterol levels, respectively, in the *stratum corneum* of the skin from Black individuals when compared to the skin of Asian and White subjects. With regards to pH, Berardesca and others observed a decrease in surface pH in the medial layers of the *stratum corneum* of Black individuals that was not observed with the White volunteer’s skin, while no difference in surface pH was observed across groups at the baseline. In contrast, Young et al. observed a significantly higher surface pH of Black *vs.* White individual’s skin at the baseline. Overall, potential differences in the *stratum corneum* physiology and structure across race are derived from a small number of studies, with a limited number of research subjects. Expanding these studies to include more volunteers and racial groups, as well as analyses of sun-exposed and sun-protected regions of the body and inclusion of skin pigmentation as a biological study variable, will be critical for a comprehensive understanding of the influence of race on the outermost layer of the human skin.

### 2.2 Transepidermal water loss

One of the main functions of the human skin barrier is the “inside-out” prevention of water loss from the body (Gilaberte et al., 2016). Non-invasive and easy-to-use tools have been developed to measure transepidermal water loss (TEWL) and are increasingly accepted as a means of qualifying the skin barrier, both *in vivo* and *in vitro* (Alexander et al., 2018). These instruments typically report a flux (g/m^2^ h or mass/surface area*time) corresponding to the steady-state water vapor flux passing through the skin barrier (i.e., *stratum corneum*), as described by Fick’s first law of diffusion (Machado et al., 2010). Importantly, TEWL has been shown to correlate with the “outside-in” permeation of topically applied compounds, suggesting TEWL is relevant to dermal absorption studies (Fluhr et al., 2006).

In pursuit of elucidating potential differences in the skin barrier across race, studies have used TEWL measurements. Several studies have reported no significant differences in TEWL as a function of race (Table 1). Berardesca and Maibach (1988) and Berardesca et al. (1998) reported no difference in basal TEWL between Black and White volunteers in concurrent studies. However, the latter of the two studies reported that TEWL was significantly increased in the Black volunteer skin after three and six tape strips when compared to the White volunteer tape-stripped skin. In a study by Reed et al., healthy subjects were divided into groups based on skin pigmentation (Fitzpatrick skin type II/III vs. V/VI), as well as race (Asian and White), and skin TEWL was measured (Reed et al., 1995). Again, no significant differences in basal skin TEWL were observed across Fitzpatrick skin types or racial groups. A more recent study categorized subjects based on Fitzpatrick skin type (II/III or V/VI) and further included an African group of people with albinism (a genetic disorder that results in a significant reduction in skin pigmentation). This inclusion uniquely allowed for the comparison of skin pigmentation and barrier independent of donor race (Voegeli et al., 2015). Under this creative experimental design, the authors reported no significant differences in basal TEWL when comparing the Black (V/VI) and White (II/III) individual groups but did find differences between the two African groups, with TEWL found to be significantly higher in the group of people with albinism compared to the Black subject group. This result was observed in both sun-exposed (cheek) and sun-protected (post-auricular) body sites, suggesting UV light damage alone does not explain the study findings.

Despite many studies reporting no significant differences in TEWL across racial groups, others have found significant differences. However, studies that report changes in TEWL do not show consensus in determining which racial group has a higher TEWL. A study by Wilson and others compared the TEWL of excised human cadaver skin from Black and White individuals. The authors reported that the Black subjects’ inner thigh skin had a significantly greater mean TEWL compared to that of the White subjects (Wilson et al., 1988). On the other hand, work by Warrier et al. determined that basal TEWL was higher for White subjects’ skin *vs.* Black subjects’ skin when measured *in vivo* on the individuals’ cheeks and legs (Warrier et al., 1996). Although many other studies reporting TEWL across racial and skin phototypes exist (reviewed in depth recently (Peer et al., 2022)), there is still a lack of a consensus as to how race or skin pigmentation relates to TEWL. As with many studies in this context, larger and more diverse cohorts, as well as better standardization of the categorization of study subjects (e.g., based on racial classification, anatomical regions, UV light exposure, age, and sex), are needed to fill this knowledge gap.

### 2.3 Compound permeation

Although dermal absorption studies are common practice in safety assessment, few studies have investigated compound permeation with the skin from donors categorized by race/ethnicity and skin pigmentation (Table 1). In a report by Williams and others, a small cohort of healthy subjects was exposed topically to two standardized formulations of nitroglycerin and blood was analyzed for nitroglycerin and its metabolites over 24 h. Although tangential to the primary objective of the study, the authors did report that the Black subjects had lower circulating levels of nitroglycerin metabolites when compared to the White and Asian subjects combined, suggesting physiological differences in the absorption and processing of topically applied nitroglycerin across these racial groups (Williams et al., 1991). Conversely, a subsequent study by Lotte et al. set a primary objective to compare dermal absorption across racial groups. Radiolabeled caffeine, acetylsalicylic acid, and benzoic acid were applied topically on Asian, Black, and White volunteers, and dermal absorption was estimated by urine collection and tape stripping (Lotte et al., 1993). No differences in compound permeation were observed across racial groups for each chemical tested.

In addition to consumer-applied topical drugs and cosmetics, the skin is continuously unintentionally exposed to numerous agents. Although determining all unanticipated exposures to the skin can be challenging, one example of occupational/environmental exposures of continued concern is heavy metals (Franken et al., 2015b). How skin pigmentation may influence the dermal absorption of heavy metals is of particular interest as melanin is negatively charged and capable of binding to cationic chemicals, including heavy metals (Potts and Au, 1976). Thus, one could hypothesize that individuals with higher levels of skin pigmentation could retain a higher level of heavy metals through interactions with melanin. In a study by Franken and colleagues, the permeation of potassium tetrachloroplatinate (platinum in salt form) was quantified *in vitro* through the excised abdominal skin of three Black and three White donors (Franken et al., 2015a). The authors reported a significant increase in the 24-h mean cumulative permeation of platinum through the Black donor skin when compared to the White donor skin. Although these data are limited by the relatively small sample size and lack of donor age matching across racial groups, they urge future investigation into the influence of melanin and race on the dermal absorption of heavy metals. Overall, the current works highlight the importance of including a diverse subject/donor cohort in dermal absorption studies. Conversely, it may also be appropriate to favor volunteers or donors based on the primary race/ethnicity of the expected users of the dermatological product undergoing assessment or their predisposition to be exposed to the concerning environmental or occupational hazard.

## 3 Future perspectives on the opportunities for reconstructed human pigmented skin models

One of the major challenges in performing non-animal, large scale dermal absorption studies is the recruitment of volunteers (*in vivo*) or the collection of excised human skin (*in vitro*). As for the latter, the excised human skin is often obtained from donors undergoing elective surgery involving skin removal, such as abdominoplasties and face lifts. Consequently, the excised human skin is inherently costly and acquiring sufficient samples can be challenging. In studies concerning race/ethnicity and the skin barrier, procuring excised skin samples from multiple human donors belonging to varying racial/ethnic groups is a major barrier to experimentation. One approach to overcome these hurdles may be the use of alternative reconstructed human pigmented skin models. In recent years, researchers have developed advanced techniques to permit the co-culture of human keratinocytes and melanocytes in a three-dimensional configuration (Hedley et al., 2002; Duval et al., 2012; Gledhill et al., 2015; Schmid et al., 2018; Zöller et al., 2019; Hall et al., 2022). These models often contain physiological ratios of keratinocytes:melanocytes and are grown at the air–liquid interface to facilitate the stratification of the epidermis and eventual *stratum corneum* development (Hedley et al., 2002; Zoio et al., 2021; Goncalves et al., 2023), giving them a similar overall structure to that of the human epidermis *in vivo*. With the production of these models, largely optimized, commercially available reconstructed human pigmented epidermal models have become more accessible (Table 2), including models incorporating melanocytes from donors of varying skin type and race. Thus, their use is no longer reserved to research groups familiar with high-level mammalian cell culture techniques and specialized equipment. Importantly, these models have been used successfully in studies aimed at investigating the biology of skin pigmentation (Itoh et al., 2020) and pigment disorders (Kang et al., 2020), and have been evaluated for use in the development of pigment modulators (Yoon et al., 2003). As such, these will likely be useful also for a variety of other applications, including IVPT.

**TABLE 2:** Examples of commercially available reconstructed human pigmented epidermis models.

Reconstructed human pigmented epidermis models		
Name	Manufacturer	Description
SkinEthic™ RHPE	Episkin	Reconstructed human pigmented epidermis consisting of normal human keratinocytes cultivated in the presence of melanocytes from phototype II, IV, or VI donors, localized in the basal layer in a 9-mm diameter transwell
Phenion^®^ epiCS-M	Phenion	Highly differentiated model of the human epidermis consisting of human keratinocytes and melanocytes cultured in a 9-mm diameter transwell
MelanoDerm™	MatTek	Normal, human-derived keratinocytes and melanocytes cultured in a 9-mm diameter transwell to form a multilayered, highly differentiated model of the human epidermis. Melanocytes from individual Caucasian, African American, or Asian donors available. Three pigmentation media containing varying levels of β-FGF and α-MSH are available

Information provided on vendor websites, as of September 2023; β-FGF, beta-fibroblast growth factor; α-MSH, alpha-melanocyte-stimulating hormone.

Despite shortcomings in the ability of reconstructed human pigmented skin models to replicate all features of the human skin (Bouwstra et al., 2021; Salminen et al., 2023), these experimental platforms do have selected advantages over the use of excised human skin for IVPT. A major benefit is the ease of obtaining samples. Depending on the location, excised human tissues may be significantly challenging to obtain under local research restrictions (Allen et al., 2010). As for the reconstructed skin models, primary human keratinocytes and melanocytes can be banked frozen, thawed, and expanded, allowing for commercially reconstructed tissue providers to produce models in a relatively quick amount of time, and with cells from multiple donors. Furthermore, in the pursuit of elucidating potential race-associated idiosyncrasies in compound dermal absorption, the highly controlled nature of these models may be of substantial benefit. For example, melanocytes from a single donor of a particular race may be incorporated into multiple constructs. These constructs can be cultured further with varying levels of melanogenesis-inhibiting or melanogenesis-promoting factors. The end result is tissue models with theoretically identical genetic make-up, only differing in their levels of pigmentation, thus providing a unique means of investigating the confounding effect of pigmentation alone. Together, these benefits ultimately support the inclusion of reconstructed human pigmented skin models in dermal absorption studies going forward. Developing rigorous methodologies for the qualification of such models will be critical to build confidence in their potential for risk/safety assessment (Salminen et al., 2023). Of note, no full thickness (epidermis and dermis) reconstructed human pigmented skin models are currently available commercially. Furthermore, in certain cases (e.g., safety assessment), the inherent biological variability of excised human skin obtained from multiple donors may be desired as it represents the general population more accurately. Developing or obtaining reconstructed human pigmented skin models comprising primary cells from multiple donors may help such models reflect some of this biological variability.

## 4 Conclusion

Assessing the rate and amount of dermal absorption of compounds of interest is a critical step in the safety assessment of topical drugs, cosmetics, and occupational and environmental exposures. As with much of the pharmacological/toxicological research to date, skin permeation studies have largely relied on White subjects or samples from White donors, limiting the relevance of these works’ findings to a racially/ethnically diverse population. The existing studies aimed at elucidating a link between race, skin pigmentation, and the human skin barrier show varying and sometimes contradictory results but encourage future work on this topic. Advances in laboratory instrumentation and techniques, including the development of reconstructed human pigmented skin models, may facilitate better permeation studies and ultimately contribute to our understanding on the role of race/ethnicity and skin pigmentation on dermal absorption.
